# Editorial: Multi-Omics Study on Gut Microbiota Related to Faecal Microbiota Transplantation

**DOI:** 10.3389/fmicb.2022.944879

**Published:** 2022-06-22

**Authors:** Jingtong Wu, Leyi Wang, Yanling Wei, Jialiang Yang, Zeyou Chen, Pei Hao, Yinyin Lv, Min Wang, Feng Liao, Longgang Chang, Yanmin Liu, Zhangran Chen

**Affiliations:** ^1^Department of Gastroenterology, Zhongshan Hospital Affiliated to Xiamen University, Xiamen, China; ^2^Veterinary Diagnostic Laboratory, College of Veterinary Medicine, University of Illinois, Urbana, IL, United States; ^3^Department of Teaching Support, Army Medical University, Chongqing, China; ^4^Geneis (Beijing) Co., Ltd., Beijing, China; ^5^College of Environmental Science and Engineering, Nankai University, Tianjin, China; ^6^Inner Mongolia Shuangqi Pharmaceutical Co., Ltd., Huhhot, China; ^7^Shenzhen Wedge Microbiology Research Co., Ltd., Shenzhen, China

**Keywords:** gut microbiota, faecal microbiota transplantation, probiotics, disease pathogenesis, COVID-19, metabolic disorders

With the advancement of multi-omics technology and experimental animal models, researchers have gained extensive information regarding the varied disease types associated with gut microbiota dysbiosis, and the causal role of gut microbiota in disease progression. As an emerging disease intervention way, faecal microbiota transplantation (FMT) has consistently demonstrated its potential in other diseases such as inflammatory bowel disease (IBD), irritable bowel syndrome (IBS), slow transit constipation, hepatic encephalopathy, autism, and metabolic syndrome more than recurrent *Clostridium difficile* infection (rCDI). However, many points remained to be further revealed in the FMT procedures. For example, it is necessary to screen and determine the healthy microbiome of stool donors to monitor the safety and efficacy of FMT. It is also important to reveal the mechanism for the efficacy of adopting FMT, which may supply a better footprint for the precise application of FMT. It is also worthy to note that other forms of microbiota transplantation besides FMT, are also promising.

This current Research Topic brings 11 references together summarizing the recent developments covering, the potential relevance of gut dysbiosis in the progression of diseases, technology and practical application of FMT.

Regarding the potential relevance of gut dysbiosis in the progression of diseases, six articles in this Research Topic individually mentioned the association of gut microbiota with drug resistances, appendectomy, age-related macular degeneration (AMD), encephalopathy caused by microbial infection, neonatal health, and obesity. Previous research showed that resistant bacteria displayed metabolic slowdown, while the molecular mechanism remains poorly understood. Through comparative proteomics analysis, Shen et al. identified nine genes involved in metabolism pathways significantly associated with MICs of amikacin/cotrimoxazole, which suggests that alteration of the metabolic network was directly correlated with antibiotic resistance. Increasing evidence has revealed that the human appendix plays important biological roles in regulating the intestinal immune system and microbiome. Cai et al. explored the alterations of gut bacterial and fungal communities associated with appendectomy in 60 subjects and found that the effects of appendectomy on the fecal fungal community are more marked and durable than on bacteria. Sepsis-associated encephalopathy (SAE) is defined as diffuse brain dysfunction without the central nervous system (CNS) infection in sepsis patients. Zhao et al. investigated the predictors associated with hospital mortality in patients with SAE and established a comprehensive visual predictive nomogram of hospital mortality, which performed better than the SAPS II with a higher net benefit. Li et al. characterized fecal microbiome and metabolomics profiles in a mouse model of laser-induced Choroidal neovascularization (CNV) and identified that Lachnospiraceae_UCG-001 and Candidatus_*Saccharimonas*, were strongly correlated with altered fecal metabolites, which might help develop novel therapeutic strategies of nAMD. Modulating the gut microbiota may be an effective strategy for alleviating obesity and related metabolic disorders. Xin et al. developed a feasible freeze-thaw pretreatment protocol to improve the extraction of microbial DNA from meconium, which would help researchers that aim to investigate the gut microbiota characteristics associated with neonatal diseases. Zou et al. evaluated the anti-obesity effects of ginsenoside Rb1 in HFD-fed mice and found the multiscale mechanisms that might account for the therapeutic effect of Rb1 against obesity. Due to the accumulating data, it is necessary to conduct computational methods for potential microbe–disease association prediction, Yang et al. constructed a comprehensive microbe–disease network by integrating known microbe–disease associations from three novel large-scale databases and extended the random walk with restart (RWR) to the network for prioritizing candidate disease-related microbes. The results suggested that it is an effective method for prioritizing novel disease-related microbes, thereby aiding our understanding of disease pathogenesis.

The selection of reliable healthy donors is a critical success factor of FMT while screening and determining the health status are involved with assessments, including health questionnaires, clinical evaluation, stool testing, blood testing, and gut microbiota test. Yu et al. developed an artificial intelligence (AI) model to identify patients who were positive for COVID-19 according to the results of the first CT examination after admission and predict the progression combined with laboratory findings, which might help efficiently exclude those subjects with COVID-19 for donor check. Although FMT is applied in constipated patients, yet the underlying mechanism remains unclear. Zhang et al. evaluated the clinical efficacy and gut microbiota remodeling ability of FMT and found the efficacy of FMT for treating constipation might be correlated with the abundance of key bacteria such as *Fusicatenibacter* and *Paraprevotella*, and butyrate production. Intestinal dysbiosis, which refers to loss of microbiome diversity and structure homeostasis in the intestine, has been proven to be associated with premature infant necrotizing enterocolitis (NEC). Lin et al. found that NEC is characterized by metabolism dysregulation, FMT and sulperazone combination treatment showed the highest benefits for the NEC. Inspired by the similarity of the intestinal and vaginal microbiota, the success of FMT inspired vaginal microbiota transplantation (VMT) proposed for the treatment of vaginal dysbacteriosis. Safe, standard, and efficient VMT will bring new hope to patients with gynecological diseases and have a good prospect of application (Han et al.).

Overall, this Research Topic provides readers with the potential relevance of gut dysbiosis in the progression of diseases, technology and practical application of FMT. However, the current Research Topic has a few limitations. Half of the accepted papers are still involved in investigating the relationship between host disease and gut microbiota, which somewhat deviate from the original intention to include more FMT-related Research Topics. And there seems to be few deep discussion concerning the mechanism of how FMT play the roles. More clinical research should be carried out to support the new therapeutic strategies targeting gut microbiota through FMT. Metagenomics, metabolomics, cultivating omics, and other omics, combined with high-throughput analysis are needed to identify the exact cross-links between host disease and gut microbiota associated with FMT.

## Author Contributions

JW, PH, YLv, MW, FL, LC, YLi, and ZC wrote and revised this article. LW, YW, JY, and ZC help co-edit the Research Topic. All authors made a substantial, direct, and intellectual contribution to this work and approved it for publication.

## Funding

This work was supported by grants from the National Natural Science Foundation of China (Grant No. 81900541).

## Conflict of Interest

JY was employed by Geneis (Beijing) Co., Ltd. MW, YLi, and ZC were employed by Inner Mongolia Shuangqi Pharmaceutical Co., Ltd. FL, LC, and ZC were employed by Shenzhen Wedge Microbiology Research Co., Ltd. The remaining authors declare that the research was conducted in the absence of any commercial or financial relationships that could be construed as a potential conflict of interest.

## Publisher's Note

All claims expressed in this article are solely those of the authors and do not necessarily represent those of their affiliated organizations, or those of the publisher, the editors and the reviewers. Any product that may be evaluated in this article, or claim that may be made by its manufacturer, is not guaranteed or endorsed by the publisher.

